# Notes Relating to the Extirpation of the Parotid Gland

**Published:** 1860-10

**Authors:** Daniel Brainard

**Affiliations:** Professor of Surgery in Rush Medical College


THE CHICAGO
### MEDICAL JOURNAL.

VOL. III. October, 1860. NO. 10.

### Original Communications.

### ARTICLE 1.

### NOTES RELATING TO THE EXTIRPATION OF THE PAROTID GLAND.

BY DANIEL BRAINARD, M. D., PROFESSOR OF SURGERY IN RUSH MEDICAL COLLEGE.

The discussion of the question of the possibility and utility of extirpation of the parotid gland will seem at first sight to imply a degree of ignorance on the part of the profession in the region in which it occurs imcompatible with a respectable degree of anatomical and surgical knowledge. Nevertheless, here, as elsewhere, it is rather the result of personal objects than of scientific zeal, raised for the purpose pulling down a school or an individual, rather than to establish any new doctrine in surgical practice. Hence I might be expected to pass it by in silence, but the discussion of any question of science is so much more agreeable than the combatting of stale notions relating to medical politics, that I choose to regard it in a serious light, and treat it is as if we were still at a period when the common carotid artery had never been tied, nor the upper and lower jaw been removed.

Prof. Granville Sharp Patteson, in a lecture delivered January 22d, 1833, p. 9, says: "If after I shall have shortly stated the facts which the progress of surgery has, within the last few years furnished, to establish the practicability and safety of extirpating a diseased parotid gland, the possibility of performing this operation should be doubted even by a junior student, I shall only say that his case is hopeless."

Professor Mutter, in his Notes to Liston's Surgery, 1846, p. 311, says: "During my recent visit to Europe being anxious to ascertain the estimate placed upon the measure by the best authorities there, I made the subject one of diligent inquiry. I discovered that much difference of opinion existed as to the utility of the operation, but I found none who doubted the possibility of its execution."

Vidal says, "The question (of the possibility of the extirpation of the parotid gland) in my judgment, need not be discussed at the present day." Traite de Pathologie Externe, vol. 4, p. 71.

Prof. Gross, in his recent work on surgery, says it is folly to deny the possibility or the propriety of extirpating the parotid gland, at the present day.

Notwithstanding these opinions, there are still those who persist in discussing this opinion, as if had never been settled; and recently the surgical and anatomical departments of the Lind University have chosen this as the point of attack upon the veracity and professional skill of the writer of this article.

Without, therefore, taking time and space, which might better be devoted to other points, I propose to lay before the profession those facts well known and accessible to all, and of which no surgeon should be ignorant, showing the perfect practicability of the operation.

I do not propose to discuss its utility except incidentally. There are some who dispute the utility of removing the female breast for cancer. The general practice of the profession is against them, although their reasons are strong, quite as much so as those which could be urged against the same operation on the parotid gland.

The question of its removal is one of indication only.

There are many cases in which the gland is involved in which no operation should be thought of. I have myself seen two cases of the kind in which no operation was advised. I certainly should hesitate to undertake the operation in cases so formidable as those in which Profs. Goodlad, Carmichael, Klein, McClellan, and others, operated successfully.

But in other cases where the disease is more limited, the operation, although formidable, is by no means excessively difficult in its mechanical execution. Cases of this kind are sometimes met with, and they are described by Nelcton as the most common form of cancer of the gland.

"The tumor remains for a long time small, indolent, and of a stony hardness; it is fixed as if wedged in the parotid notch. It presents no inequalities on the surface, and no alteration of the skin. It is developed from above downward, and its greatest diameter is transverse."

"This tumor increases but slowly, and it is but rarely, according to Boyer, that it quickly acquires a large size, and that it gives rise to the sensations peculiar to cancer." *Elements de Pathologie Chirurgicale*, tona 2, p. 725.

The objections to the reports of the removals of this gland date from an early day, before the ligature of the carotid artery was thought of, and when the danger of wounding the external carotid was sufficient to deter surgeons from undertaking an operation. They are mostly anatomical, and refer to the deep situation and important relations of the organ. Experience has sufficiently demonstrated the fallacy of such objections. There is no wound fatal to life necessarily made in removing the parotid gland.

Critics have objected that reports of this operation often state that there is little hemorrhage, which, it is alleged, is inconsistent with the idea of its extirpation. Now the principal artery involved in tumors of the gland when of small size is the continuous trunk of the external carotid, which divides higher up into the temporal and internal maxillary arteries. Owing to anomalies this may be deficient, or of small size, but even if of its usual dimension, it does not necessarily give great hemorrhage; firstly because the surgeon, knowing its situation, may secure it by ligatures before dividing; secondly, because an artery of that size, when torn across, give little or no blood. This was noticed by Dr. George McClellan in one of his cases. If any not familiar with such operations doubt of this let them refer to the effect of torsion of the arteries or to those cases of which four or five are now on record in which avulsion of the arm and scapula was produced by mechanical force, the subclavian and axillary arteries, giving little or no blood when thus torn across.

The amount of hemorrhage will, in such an operation depend upon the vascularity of the parts and the size of the tumor. I have removed about twenty tumors from the region about the angle of the jaw, including several of portions of the jaw itself. Of these, two only were for disease of the gland.

In some of these operations the bleeding was profuse, much more so than in the two for removal of the gland itself. McClellan states the same as true of his experience. He removed the gland eleven, and other tumors situated in connection with it, thirty times.

The manner of performing the operation also influences the loss of blood. By tying the large arteries before dividing them, and making the dissection wih a blunt instrument, large tumors may be removed without great hemorrage.

Another objection to the reports of such operation is the neglect to state that the facial nerve was divided, or that paralysis occurred. Now, if in any such operation, the face were stated not to be paralyzed, or the external carotid artery, not to be divided, I should doubt if the parotid gland could have been removed, notwithstanding it may be taken away in the healthy state on the dead subject and leave both these parts. But the neglect to give a full description of an operation does not altogether discredit it, and after the division of the nerve the paralysis gradually disappears or diminishes, probably from other nervous branches supplying its place. This has been noticed by Sir Astley Cooper and others.

Finally, it has been objected that surgeons have mistaken other glands for the parotid. It may be well to notice the distinction between the question of primary disease of the parotid, and that of its removal by operation. It might be true that the gland is not subject to become primarily diseased, and yet it might require to be removed for disease extending to it. What difference, in a purely surgical point of view, can there be between a cancerous growth originating a lymphatic gland situated within the parotid and extending to this latter, causing its absorption, and the same growth originating in the parotid and extending to the lymphatic glands? Scirrhus of the female breast sometimes originates in the skin. —Velpeau *Traite des Maladies de Sein*, p. 437.

But this in no wise changes its nature nor obviates the necessity of removing the whole gland when this has become involved. I have, in three cases of disease of the lower jaw, removed the half of it, with every vestige of both the parotid and submaxillary glands, which were perfectly involved in the disease mass.

It will be noticed that Nelaton describes scirrhus of the parotid gland as developing itself from above downwards. McCellan pointed out that in some cases of disease of the gland it gradually raised itself out of the triangular space in which it is placed, so as present fewer difficulties in removal than when in a healthy state. Dr. N. R. Smith speaks of its even becoming "pediculated." Pancoast attributes to Randolph the first observation of this fact.

In the first operation which I performed, the finger passed under the gland from below upwards with a facility which surprised me. The disease was a scirrhus of the gland.

While admitting the difficulty of distinguishing between cases of disease originating in the tissue of the gland and those extending to it. I am not prepared to believe that any well informed surgeon need be in error in regard to the parts actually removed by operation. I am in the habit, after all such operations, of examining the tissues with a magnifying glass, of having them examined with the microscope, and of carefully inspecting the whole surface of the wound for any suspicious tissue. I generally, and always unless some reason for a different course appears, defer closing the wound until the oozing of blood has entirely ceased. By thus carefully proceeding, mistakes can be, for the most part, avoided.

In the lecture referred to by a Professor in the Lind University, the opinion was advanced that the parotid, with the exception of enchondroma, is absolutely exempts from disease. This assertion was not called in question by any one except Dr. Paoli, who, in a recent No. of the *Louisville Journal*, has published a valuable article on the subject.

This is so gross an error that it ought to be at once detected by any medical practitioner, and in order to place the subject in its true light I here transcribe, from my notes for lectures, authorities sufficient to leave no doubt in the mind of any person desirous of learning the truth on the subject.

Dr. J. H. Bennett relates a case of disease originally in in the parotid gland, having all the appearance of malignancy. Mr. Syme advised against an operation. The patient died. Mr. Bennett found no cancer cells, and considered it cancroid. He also quotes a similar case from M. Sedillot, in which a tumor, having all the characteristics of malignancy, ulcerated and soon entirely destroyed the parotid gland, but as no cancer cells were found, Sedillot considered it cancroid. This also proved fatal.—On Cancers and Cancroid Diseases; Edinburgh, 1849, p. 83, *et. seq.*

Dr. Lebert (*Traite Practique des Maladies Cancereuses*, p. 703) says: "We have observed, for our own part, cancer of the parotid gland three times." "The developement, march, termination, and treatment of cancer of the parotid, are about the same that we have indicated for cancer of the lymphatic glands." "But it should should be remembered here that cancer of the parotid, in its progress, involves the jaw, and in case of extirpation all the part of the bone diseased should be taken away."—Ib., p. 704.

"The tissue of the parotid sometimes becomes, though rarely, the seat of a scirrhus degeneration." "Extirpation is the only means that we can oppose to this disease." "When the whole of the parotid is to be removed, the plan which appears to us preferable is that which was proposed by M. Begin, (Roche, Sanson, and Lenoir.—*Nouvaux Elements de*

*Pathologie Medico-Chirurgicale*, p. 115, vol. iii. Paris, 1844.)

Nelaton, in the second volume of "Pathologie Chirurgicale," page 714, *et. seq.*, gives a very full and succint review of the diseases of the parotid. Besides wounds and salivary fistulae, he describes:

1. Abcesses, which among other points of origin "may be in the salivary gland itself."

2. Concretions and salivary pouches. "These concretions," he says, "are liable to obstruct the flow of saliva so that these result sometimes in a voluminous tumor, oblong, indolent, circumscribed.

3. Cysts; of which an example is quoted from the bulletin of the Academy of Medicine, vol. 1, p. 56.

4. Hypertrophy. Of this two examples are quoted from A. Berard and Sabbatier.

5. Fibro-fatty and Melanotie.

6. Sauguine tumor.

7. Cancer. There are some cases where the parotids present a cancerous degeneration. Mekel has dissected a certain number, and Berard reports some examples in his thesis, already cited. When the gland itself is degenerated, we find usually a scirrhus instead of an enaplalous tumor. This tumor remains a long time indolent, small, and of a stony hardness.

*Treatment.*—The extirpation of the tumor is the only means of relieving the patient. Neither its considerable volume nor its adhesion to subjacent parts, nor ulceration of the skin ought to be considered absolute contra-indications to the operation.

The facts which render it probable that the gland has been entirely removed, are those of Warren, Kirby, Milman, McC'elland Preiger, Raymond, Charmichael, and Gensoul. Those which prove that the gland has certainly been removed are, according to M. Berard, those of Randolph, Smith, Lisfranc, Gensoul, Beclard, Bramberg, p. 73.

*Chelius.*—The removal of the parotid gland belongs to the most difficult and dangerous operations, as the close connection of the gland with the important neighboring parts render necessary the wounding of very important vessels and nerves, etc. P. 523, vol. 3.

Cases of the removal of the parotid gland are related by Preiger in Von Graffae and Walther's *Journal*, vol. 2, p. 454, and in *Rust's Magazine*, vol. 19, p. 303.

Barendts, ib. vol. 13, p. 159.

Schmidt, ib. p. 312.

Weinhold, *Sulzberg Med. and Chir. Gazette*, vol. 4, p. 63; 1823.

Beclard, Archies Geerales de Medicine; 1824. Vol. 4, p. 62.

Chelius, Heidleberg Clinical Annals, vol. 2, p. 2.

Kirby, J., Dublin, 1825.

In the case of Dr. Preiger, the tumor weighed two pounds and three quarters, and the operation lasted seven minutes.

In summing up this testimony on the subject in 1842, Reese, in his Notes to Samuel Cooper's Surgical Dictionary, p. 259, vol. 2, said:

"The parotid, in a scirrhus state, can be entirely extirpated."

"In Dr. Preiger's, Mr. Kirby's, and of McClellan's cases, the trunk of the external carotid was left untouched."

Mr. Goodlad's case is reported in the Med. Chir. Transactions, vol. 7, p. 112, and vol. 8, p. 582: "The disease extended from the external canthus of the eye above, to within three quarters of an inch of the clavicle below, and some idea of the depths of its attachments may be formed, when it is known that the whole of the parotid gland is involved in it." "*The parotid gland was entirely removed.*—Samuel Cooper's Dictionary, p. 273, vol. 1.

In vol. 2, p. 101, Transactions King's and Queen's College of Physicians, Dublin, 1818, is reported Mr. Charmichael's case, "The tumor was very large, involved the parotid gland, and connected with the transverse process of the atlas, the basis of skull, the meatus auditorius, mastoid process, and the angle of the jaw." The case was successful.

Klein operated on a women of seventy, "The swelling extended from the ear to the shoulder." "The internal carotid artery and the par vagum were left quite bare." "The bleedin was very inconsiderable.". "The operation was so successful that in the beginning of the third week the wound was healed.—Ib.

Beclard's case was for a scirrhus of the parotid alone. Samuel Cooper says the following inferences are deduced from this case: 1. "The reality of the scirrhus of the salivary glands is confirmed." 2. "The possibility of removing the parotid demonstrated."—Ib. p. 274.

This case proved fatal, and an examination was made after death, so that no dispute concerning the operation being the entire removal of the parotid gland could arise.

There is no surgeon who did so much to establish the operation of extirpation of the parotid gland as a legitimate one in surgical practice, as the late Dr. Geo. McClellan, of Philadelphia. Much of the acrimony which has been introduced into this discussion has no doubt arisen from that controversy in a great measure personal, and foreign to the purely scientific question, into which he was drawn by the attacks upon his character as a surgeon. These attacks, prompted by the same motives, are still occasionally made upon those who propose the operation.

Dr. McClelland says, (Principles and Practice of Surgery, p. 352,) "I have extirpated *eleven entire parotids*, in various conditions of organic disease, and only one of my patients died in consequence of the operation."

"The next case which occurred to me I turned over to my son, who successfully removed an enormous tumor, involving the whole parotid, an immense mass of lymphatic glands, extending down the neck, with the common carotid artery, the internal jugular vein, the par vagum, spinal accessary, and portio dura nerves. The patient, George Andrews, of Washington, Pa., went home in four weeks, with the wound entirely healed."

This case is worthy the attention of those who think the relations of the gland to the great vessels are such as to render an operation impracticable, even when it does not touch them.

2

But this appears to be the only case of operation on record in which those vessels were involved. In one of Dr. McClelland's cases the external carotid did not require a ligature. "Before he proceeded farther he isolated the continued trunk of the external carotid just as it was entering the tumor together with the descending veins, which accompanied it, and, instead of cutting them across, in the usual manner, he tore them out from the body of the tumor with the thumb and fore-finger." The vessels bled for a moment only, and ceased without a ligature.—Ib., p. 334.

In one of Dr. McClelland's cases, (Mr. Socher,) death took place from an abdominal disease, eighteen months after the operation, and the body was carefully examined. No trace of the parotid gland could be detected.—Lecture on the Parotid Gland, by G. S. Pattison, 1833, p. 13.

The case of John Bell is related in his Principles of Surgery, New York, 1810, p. 507, *et seq.*: "The tumor, of the size of a goose,s egg, was once removed with about half the gland. It returned and Mr. John Bell removed the whole of it.

Liston (Lectures on the operations of Surgery, Philadelphia, 1846, p. 308,) says, on the subject of of Diseases of the Neck: "Tumors of this region occur in different aspects and corners of it. You meet them at the upper part of the neck aud at the angle of the jaws. They sometimes arise from a degeneration of the salivary glands; but that is not a common occurrence." "Sometimes they are very firmly fixed, but after all they come out easily enough."

Colles, who was one of those who, for anatomical reasons, believed the removal of the gland impossible, (was it because Charmichael had performed the operation ?) says, under the head of Salivary Glands: "They are sometimes the seat of scirrhus."—Lectures on the Theory and Practice of Surgery, Philadelphia, 1845; p. 107.

Rokitansky enumerates, among the diseased conditions observed by himself in the parotid, inflammation, cysts, fibrous, cartilaginous, and osseous growths, etc.

"Carcinomatous disease occurs in the parotid and salivary glands, and especially in the parotid, in the shape of scirrhus and medullary cancer.—Path. Anat., vol. ii, p. 141.

Warren gives a minute and accurate description of the tumors of this gland:

"The parotid gland is the seat of two species of induration, one in the lymphatic glands situated about it, which is quite common; the other a genuine induration of the gland itself, which is quite rare."

"The induration or scirrhus of the parotid gland begins deeply in the space between the branch of the jaw and the mastoid process. Its confined situation renders it painful at an early period. Being confined on the sides by bone, it extends outwards and downwards, assuming a conical form in all the cases which I have seen. This tumor is of a scirrhus hardness." "Ulceration does not take place in a long series years."

"The scirrhus state of the parotid being incurable by remedies, a surgical operation is the only resource. An operation requiring some degree of skill, coolness, and a knowledge of anatomy."—On Tumors, p. 286, 287.

On the subject of these diseases, Dr. Gross says: "All the salivary glands are not equally liable to morbid alterations. The parotid is infinitely more frequently affected than the others." "Parotitis, under favorable circumstances, may terminate in suppuration. Of this I have witnessed a considerable number of cases, in two of which an opportunity was offered of making an autopsic inspection." Gangrene has been known to seize on the parotid." "The salivary glands, especially the parotid, are subject to atrophy." "True scirrhus is occasionally met with in the parotid, as are also encephaloid and melanosis."—Path. Aut., p. 518, 519.

Baron J. D. Larry removed the parotid gland for cancerous disease, March 19, 1841. Numerous enlarged lymphatic glands were discussed by a long course of treatment when "there remained the parotid tumor of the form and size of a large hen's egg, deeply situated between the ear and the ramus of the jaw. It was soft at the center, hard at the circumference, and slightly movable.

On the 70th day the patient was entirely recovered, the sensibility and the movements of the side of the face, which had been destroyed at first, were restored in part.

In concluding the report of this and a case of removal of the submaxillary gland, M. Larrey remarks "From these two examples of complete success obtained by extirpation of salivary glands, (the parotid and submaxillary) we may conclude that this extirpation is practicable, and may be performed without danger to the life of the patient."—*Memoire sur L'Extirpation des Glands Salivaire*, Paris, 1841; pages 21 and 22.

From these authorities alone, (and many others might be cited,) we may safely conclude that the parotid gland is subject to disease, and that its removal in favorable cases is both possible and advisable.

I add a list of nearly one hundred operations of the kind, omitting above fifty others, concerning which my information is too limited to enable me to judge of their value. Some of those inserted have been objected to on grounds which appear insufficient. Of those cited from American sources, I see no reason to doubt the correctness of a single one. I have omitted eleven cases of this kind on account of insufficiency of the details:

| | | | | | |
|---|---|---|---|---|---|
| Chelins | 8 | | Brought forward | 20 | |
| Preiger | 1 | 1826 | Samuel White | 1 | 1808 |
| Barendts | 1 | | Geo. McClellan | 11 | |
| Schmidt | 1 | | Klein | 1 | |
| Weinhold | 1 | | J. H. B. McClellan | 1 | |
| Beclard | 1 | 1823 | Gensoul | 2 | 1828 |
| Goodlad | 1 | | Seibold | 1 | 1781 |
| Charmichael | 1 | 1818 | Scultetus | 1 | |
| Lisfranc | 1 | 1826 | Gooch | 1 | |
| Manfredini | 1 | | Behr | 1 | |
| Idrae | 1 | | Palfin | 1 | |
| Kirby | 1 | | Verduin | 1 | |
| Busche | 1 | 1827 | Acrel | 1 | |
| Carried forward | 20 | | Carried forward | 43 | |

| | | | | |
|---|---|---|---|---|
| Brought forwaad | 43 | Brought forward | 67 | |
| Astley Cooper | 2 | L. Herminier | 1 | |
| Dav. Prince | 1 | Heyfelder | 1 | |
| J. C. Bradbury | 1 | Berret | 1 | |
| J. C. Warren | 1 | M. N. Fouthern | 1 | |
| Mott (several) | 3 | A. Magri | 1 | |
| N. R. Smith | 1 | Hendricks | 4 | |
| Randolph | 1 | Eulenberg | 1 | |
| Roux | 1 | Stedman | 1 | |
| Cordes | 1 | Awll | 1 | |
| Warren (Elder) | 1 | Eckstrum | 1 | |
| J. D. Larrey | 1 | Anisaux | 1 | |
| J. C. Bradbury | 1 | McGregor | 1 | |
| Van Sweiten | 1 | Widmer | 1 | |
| Roonhuysen | 1 | Ohle | 1 | |
| Gottfried | 1 | Dieffenbach | 1 | |
| Errhart | 1 | Wallman | 1 | 1828 |
| Scharschmidt | 1 | Bendz | 1 | 1838 |
| Soucramp | 1 | Naegle | 1 | 1830 |
| J. B. Seibold | 1 | J. Bell | 1 | |
| Moulinia | 2 | Brainard | 2 | |
| Carried forward | 67 | | 91 | |

